# Optimization of probiotic therapeutics using machine learning in an artificial human gastrointestinal tract

**DOI:** 10.1038/s41598-020-79947-y

**Published:** 2021-01-13

**Authors:** Susan Westfall, Francesca Carracci, Molly Estill, Danyue Zhao, Qing-li Wu, Li Shen, James Simon, Giulio Maria Pasinetti

**Affiliations:** 1 Department of Neurology, Icahn School of Medicine at Mount Sinai, New York, NY USA; 2 Geriatric Research, Education and Clinical Center, James J. Peters Veterans Affairs Medical Center, Bronx, NY USA; 3 Department of Plant Biology, Rutgers University, New Brunswick, NJ USA

**Keywords:** Microbiome, Symbiosis, Biotechnology, Gastrointestinal models, Machine learning

## Abstract

The gut microbiota’s metabolome is composed of bioactive metabolites that confer disease resilience. Probiotics’ therapeutic potential hinges on their metabolome altering ability; however, characterizing probiotics’ metabolic activity remains a formidable task. In order to solve this problem, an artificial model of the human gastrointestinal tract is introduced coined the ABIOME (A Bioreactor Imitation of the Microbiota Environment) and used to predict probiotic formulations’ metabolic activity and hence therapeutic potential with machine learning tools. The ABIOME is a modular yet dynamic system with real-time monitoring of gastrointestinal conditions that support complex cultures representative of the human microbiota and its metabolome. The fecal-inoculated ABIOME was supplemented with a polyphenol-rich prebiotic and combinations of novel probiotics that altered the output of bioactive metabolites previously shown to invoke anti-inflammatory effects. To dissect the synergistic interactions between exogenous probiotics and the autochthonous microbiota a multivariate adaptive regression splines (MARS) model was implemented towards the development of optimized probiotic combinations with therapeutic benefits. Using this algorithm, several probiotic combinations were identified that stimulated synergistic production of bioavailable metabolites, each with a different therapeutic capacity. Based on these results, the ABIOME in combination with the MARS algorithm could be used to create probiotic formulations with specific therapeutic applications based on their signature metabolic activity.

## Introduction

The gut microbiota is a complex ecosystem of bacteria, fungi and archaebacteria living in symbiosis with humans. The gut microbiota provides an plethora of beneficial functions^1^ including selective fermentation of dietary carbohydrates and polyphenols producing a metabolome that can elicit therapeutic benefits^2^. Disturbance of gut microbial populations or their metabolism, known as dysbiosis, introduced due to age, pharmaceuticals, antibiotics, diet or stress contribute to chronic disease development. Probiotics can reverse dysbiosis by supporting a balanced gut microbiota and stimulating beneficial metabolite production collectively providing disease resilience^2–4^. We previously demonstrated that two phytochemicals, dihydrocaffeic acid and malvidin-3′-O-glucoside, derived from a grape-derived polyphenol-rich prebiotic promote resilience to stress-induced depression by modulating brain synaptic plasticity and peripheral inflammation, respectively^5^. Further, we verified that the beneficial effects of this prebiotic depends on the composition of the gut microbiota^6^. These beneficial phytochemicals are among a battery of beneficial metabolites produced by the gut microbiota whose production and bioavailability could be maximized with systematically designed probiotic therapies.

A major obstacle in the study of the gut microbiota and its metabolome towards the development of probiotic therapeutic products is the scarcity of accurate, yet practical, models of the human gastrointestinal (GI) tract. The GI tract consists of distinct regions defined by pH, substrate availability, cellular composition and transit time, which alter the composition and function of the autochthonous microbiota. In vitro models of the human GI tract have been developed to recapitulate the critical physiological aspects of the gut microbiota and its interaction with the host. Unfortunately, many of these systems are limited by size, technical complexity or uniqueness making their usability limited. The simplest models are the batch-incubator chemostat systems containing a single fermentator without dynamic control of pH or liquid aspiration^7^. Multi-chamber systems including the mucosal Simulated Human Intestinal Microbial Ecosystem (M-SHIME)^8,9^ or the RoboGut^10^ have the added benefit of modelling discreet conditions along the GI tract. Although physiologically relevant, these multi-chamber systems are large, require long stabilization times and have limited ability to run samples in parallel. Perhaps the most complex and integrated system is the TNO (gastro-) intestinal models of digestion and absorption^11^; however, these systems require customized design and programming that cannot be easily adapted into lab environments. Finally, miniaturised in vitro models such as gut-on-a-chip^12^ have been designed that can even recreate the anoxic–oxic interface defining the mucosal bacteria’s microenvironment^13^. The limitation of these smaller systems is the difficulty in establishing a stable gut microbiota with the same complexity as larger systems and since the volume is so low, studying the gut microbial populations or metabolites using standard microbiological techniques is nearly impossible.

To dissect the complexity of the gut microbiota, machine learning and artificial intelligence have become important computational tools to discover trends and synergies in large data sets that are otherwise eclipsed by conventional analytical techniques. These algorithms are highly adaptable, trainable and designed to account for limited or missing information in line with the practical confines of preclinical and clinical research^14^. Some groups have begun testing computational tools to assess associations between microbiome metagenomic datasets and disease phenotypes^15,16^; however, machine learning tools have not been used to predict the gut microbiota’s metabolome towards therapeutic optimization of probiotic formulations.

To address this gap in the availability of practical tools to optimize probiotic formulations in vitro*,* this study introduces the ABIOME (A Bioreactor Imitation of the Microbiota Environment)—a modular yet dynamic artificial model of the human GI tract supporting the complex human gut microbiota. Due to its modularity, the ABIOME is easily procured and does not require customized programming: a unique feature among available artificial GI models. Using the novel ABIOME, the authors built the hypothesis that a multivariate adaptive regression splines (MARS) model could be developed to predict the human gut microbiota’s metabolome following supplementation with probiotic combinations and a polyphenol-rich prebiotic previously shown to have therapeutic efficacy^5^. This machine learning algorithm can predict potential synergisms between a finite number of tested probiotic combinations that may be effective at generating bioactive metabolites with therapeutic properties.

## Results

### Validating the ABIOME

**Figure 1:**
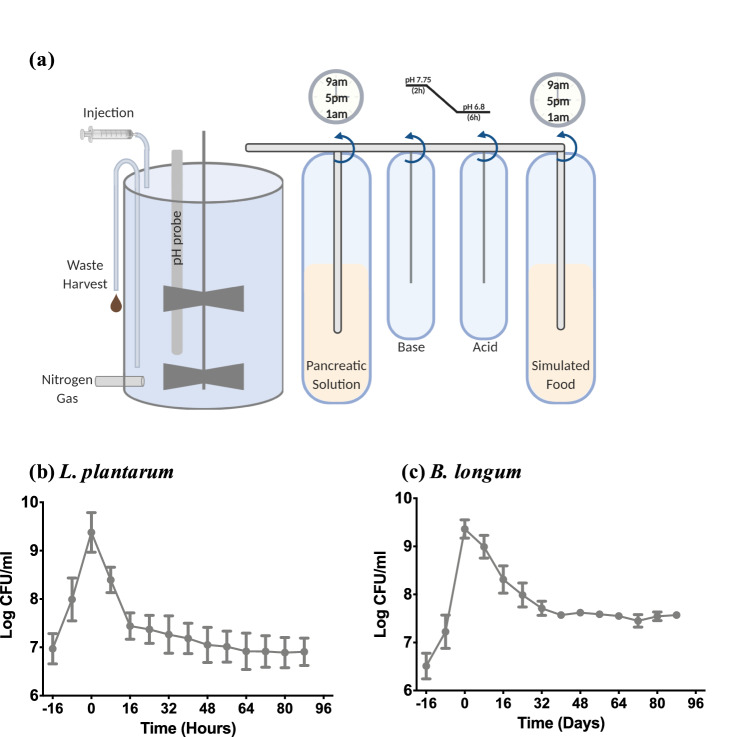
A novel ex vivo gastrointestinal model can maintain typical commensal cultures. A schematic of (**a**) A Bioreactor Imitation of Microbiota Ecosystem (ABIOME) shows the main bioreactor unit being connected to a computer controlled pumping system from each of the input solutions: pancreatic solution, base, acid and simulated “food”. The food and pancreatic solution inputs are controlled as a thrice-daily feed cycles while the acid and base maintain the pH set-points of the bioreactor at a pre-set ramp rate. With the feeding cycles, there is a thrice daily “waste harvest” where a portion of the bioreactor contents is removed to circulate wastes. The bioreactor unit is kept under strict anaerobic conditions via constant nitrogen sparging while the contents are constantly agitated by the stirring system. Using this system of feeding and pH control, monocultures of (**b**) the aerobic *L. plantarum* and (**c**) anaerobic *B. longum* reached a steady-state level after several hours of incubation. Cultures were inoculated in the food source at − 16, − 8 and 0 h, emulating the thrice daily feeding, after which the maintenance of the cultures was observed for 88 h. Cultures were enumerated with serial dilutions and plating on MRS and MRS-cysteine plates, respectively, following calculation of the number of colony forming units (CFU) per milliliter. Each time point represents *n* = 3 independent trials  +/− SEM.

To understand if the conditions, temperature, pH cycle, media or pancreatic conditions were suitable for the growth of bacteria in the ABIOME (Fig. 1a, Supp. Fig. 1), the bioreactor was inoculated with a monoculture of either the aerobic (*Lactobacillus plantarum*) or anaerobic (*Bifidobacteria longum*) bacterium. The bacteria was dosed into the bioreactor through the normal feeding cycle every 8 h at a concentration of 1 × 10^10^ colony forming unit (CFU)/meal resulting in accumulation of bacterial CFUs at − 16, − 8 and 0 h before the ABIOME was allowed to run without inoculation. Both *L. plantarum* (Fig. 1b) and *B. longum* (Fig. 1c) reached a maximum concentration of 9.5 log CFU/mL after three consecutive doses. *L. plantarum* levels rapidly dropped over the next 16 h stabilizing at 7.0 log CFU/mL after 24 h. *B. longum* concentrations reduced more conservatively reaching a steady state of 7.8 log CFU/mL after 32 h.

### The ABIOME supports complex human microbiome populations

**Figure 2:**
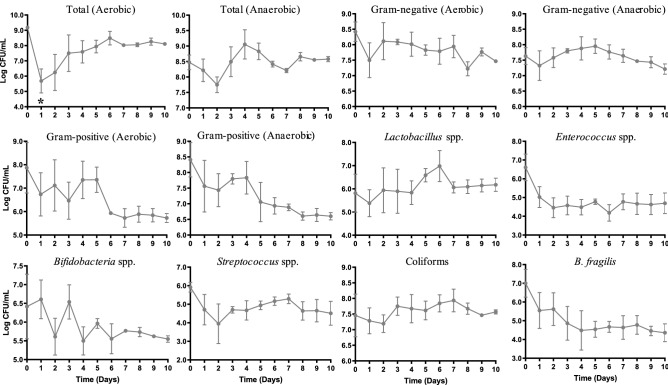
Viable cell counts following fecal inoculation. Viable cell counts of 12 groups of commensal gut microbiota were assessed for 10 days following fecal inoculation on selective agar plates. Plating of serial dilutions allowed the number of colony forming units (CFU) to be determined each day. Each time-point represents *n* = 3 independent trials  +/− SEM. Variability over time was conducted with a one-way ANOVA using Kruskal Wallis analysis where *p < 0.05. −

The complete human microbiota offers new challenges to an artificial GI system as populations of bacteria compete for resources and space. To demonstrate that the ABIOME can support a complex human microbiota culture, 10% fecal slurries were inoculated into the bioreactor at day 0 and variations in viable gut microbiota populations were monitored using agar exclusion plating for the following 10 days (Fig. 2). After an initial reduction in the total aerobic populations from 9.1 CFU/mL at day 0 to a minimum of 5.7 CFU/mL at day 2 (*p* < 0.015), stabilization of total aerobic populations was reached by day 5 at 9.0 ± 0.2 CFU/mL. Anaerobic populations displayed no significant variation over the 10 days of stabilization (F(10,22) = 1.58, p > 0.05) with an overall steady state at 8.5 ± 0.1 CFU/mL. Neither the aerobic (F(10.22 = 0.95, p > 0.05) nor anaerobic (F(10,22) = 0.78, p > 0.05) gram negative groups displayed variability 10 days following inoculation and reached similar overall steady-state levels at 7.8 ± 0.3 and 7.6 ± 0.2 CFU/mL, respectively. Similarly, neither the aerobic (F(10.22 = 0.1.30, p > 0.05) nor the anaerobic (F(10,22) = 1.87, p > 0.05) gram positive groups displayed variability 10 days following inoculation. Nevertheless, there was greater variability between trials from days 1 through 6 for the gram positive aerobic group reaching a steady state by day 7 at 5.7 ± 0.4 CFU/mL. The gram positive anaerobic group also showed smaller variability between trials beginning at day 7, plateauing at 6.9 ± 0.1 CFU/mL (Fig. 2).

At the genera level, aerotolerant *Lactobacillus* spp. had no variation over time (F(10,22) = 0.49, p > 0.05) yet the variability between trials was considerable for the first 7 days, reaching a more consistent stabilization by day 8 at 6.1 ± 0.3 CFU/mL. The *Enterococcus* genera reached a consistent quantity at day 3 of 4.6 ± 0.5 CFU/mL with no variability for the remaining 8 days (F(10.22) = 1.57, p > 0.05). *Bifidobacteria* spp., an anaerobic genera of bacteria, had a high variability between trials and consequently, a stabilization period was reached by day 6 at 5.5 ± 0.4 CFU/mL. The *Streptococcus* genera also had a high inter-trial variability for 4 days (F(10,22) = 0.82, p > 0.05), after which a stable level of 4.9 ± 0.2 was reached by day 5. The coliforms group, including *E. coli*, was maintained relatively consistent between trials (F(10,22) = 1.31, p > 0.05) reaching a constant level of 7.5 ± 0.6 CFU/mL by day 5. Finally, *Bacteriodes fragilis*, a populous obligatory anaerobe and opportunistic pathogen, was found to have high inter-trial variability over the first 5 days, stabilizing at 4.6 ± 0.3 CFU/mL at day 6. Overall, the viable cell counts using agar exclusion plating shows that a complex representation of the human microbiota from fecal matter can be maintained in the ABIOME, stabilizing, on average 5 days following inoculation.

**Figure 3:**
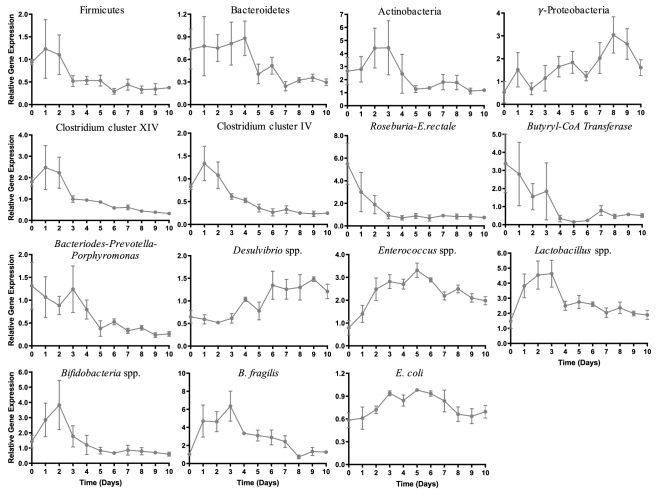
Real-time PCR quantification of 16 S V1–V3 region of gut microbiota in the ABIOME. Quantification of 16S rRNA V1–V3 regions of the gut microbiome was conducted using qPCR and gene expression relative to a reference sample and the total amount of bacteria as indicated by the 27F/519R primer set. Relative gene expression was calculated using the 2^−ΔΔCT^ method. Each time-point represents *n* = 3 independent trials +/− SEM. Variability over time was conducted with a one-way ANOVA using Kruskal Wallis analysis where *p < 0.05. −

To gain a higher resolution of bacterial groups present in the ABIOME following inoculation, real-time qPCR analysis of several key phyla and genera was conducted (Fig. 3). Quantification of the three major phyla, Firmicutes, Bacteroidetes, Actinobacteria and one class, γ-Proteobacteria, which represents 98% of the intestinal microbiota^17^, revealed different trends. Firmicutes, a mostly gram-positive phyla, slightly reduced over the first 5 days of stabilization normalizing at a relative gene expression of 0.52 ± 0.1 by day 3, a 45% reduction compared to day 0. Bacteroidetes, a phyla consisting of mostly gram-negative bacteria, reduced by 73% in relative expression reaching the stabilization of 0.32 ± 0.03 at day 7. The Actinobacteria phyla was reduced by 47.9% over the first 5 days reaching a relative expression plateau of 1.27 ± 0.23 by day 5. γ-Proteobacteria, a gram-negative class of Proteobacteria, increased by 2.3-fold over the first 5 days, however, due to the high variability between samples, a steady-state level was not reached (F(10,19) = 1.672, p > 0.05).

The *Bacteroides–Prevotella–Porphyromonas* (BPP) group consists of obligatory anaerobes apart of the normal commensal gut microbiota; however, they are common genera found in cases of infection if over expanded. The BPP group stabilized by day 5 at 0.40 ± 0.1 relative gene expression, a 72% reduction from day 0. *Delsulfovibrio* spp., is a genus of aerotolerant sulfur-reducing bacteria increased by 2.0-fold in the first 6 days reaching a constant relative gene expression of 1.34 ± 0.3. *Enterococcus* spp. is a large genus of gram-positive lactic acid producing bacteria that are facultative anaerobes. Relative expression of *Enterococcus* spp. increased over 5 days to a level 4.1-fold greater than day 0, but stabilized at a lower relative expression at 2.2 ± 0.2 at day 7. *Lactobacillus* spp. are gram-positive facultative anaerobic genera generally identified as an indicator of healthy gut microbiota. *Lactobacillus* spp. relative gene expression stabilized at 2.0 ± 0.3 relative gene expression, a 1.4-fold increase from day 0. The *Bifidobacteria* spp. is another genera of bacteria typically regarded as an indicator of a healthy microbiota that consists of gram-positive anaerobes. In the first 3 days, the relative expression of *Bifidobacteria* spp. was highly variable before stabilizing at 0.67 ± 0.09, with overall no variation over the 10 days (F(10,21) = 2.3, p > 0.05). *B. fragilis* is a highly populous gram-negative obligatory anaerobe. Despite a slight increase from a relative gene expression of 1.03 ± 0.03 to a maximum of 6.4 ± 1.5 at day 3, *B. fragilis* stabilized at a relative gene expression of 1.2 ± 0.2 by day 8, which is only a 1.2-fold increase compared to day 0. *E. coli* constitutes a major population of the γ-Proteobacteria class, is a facultative gram-negative anaerobe found in the commensal microbiota. There was little variability between samples and over time in the *E. coli* group with an overall stabilized relative gene expression of 0.77 ± 0.08.

Clostridium cluster XIVa (also known as the *Clostridium coccoides* group) is an abundant group of obligatory anaerobic, spore-forming, gram-positive bacteria including the butyrate producing *Ruminococcus, Roseburia* and *Eubacterium* spp. This group stabilized by day 6 with a relative fold change of 0.58 ± 0.03 accounting for a 68% loss compared to Day 0. The Clostridium cluster IV (also known as the *Clostridium leptum* group) is another abundant group of butyrate-producing obligatory anaerobic bacteria of which *Faecalibacterium prausnitzii* is a major constituent. The Clostridium cluster IV group stabilized at a relative fold change of 0.26 ± 0.08 at day 6, representing 30% of the total Clostridium cluster IV observed at Day 1. Among the genera constituting the Clostridium cluster XIVa group is the *Roseburia-E.rectale* genera and similar to the trend of Clostridium cluster IV group, the *Roseburia-E.rectale* genera stabilized by day 5 at 0.88 ± 0.19 relative expression, accounting for 16.1% of the relative population compared to day 0. This decrease in the prominent butyrate producers was reflected by the expression of the functional butyrate gene butyryl-Coenzyme A(CoA) CoA transferase gene, which is essential for the production of butyrate in the commensal microbes^18^. Stabilization was reached by day 8 at 0.46 ± 0.10 relative gene expression, only 13.5% of the relative expression at day 0.

### Designing optimized probiotic formulations

To streamline the design of therapeutic formulations from a battery of probiotic bacteria with unknown synergies, a multivariate regression analysis (MARS) was employed. There are two main reasons explaining how probiotics’ contribution to metabolite production is more complex than a linear combination. First, there is an interaction between probiotics and the overall composition of the gut microbiota. Second, the metabolic byproducts from one bacteria, probiotic or commensal, can be used as a fermentation precursor for another group of bacteria creating potential synergies in metabolite production. Instead of testing a significant number of combinations of potentially synergistic probiotics, which is often practically impossible, machine learning algorithms can predict based on a relatively small dataset how probiotics may work together synergistically to maximize the production of therapeutically relevant metabolites.

**Figure 4:**
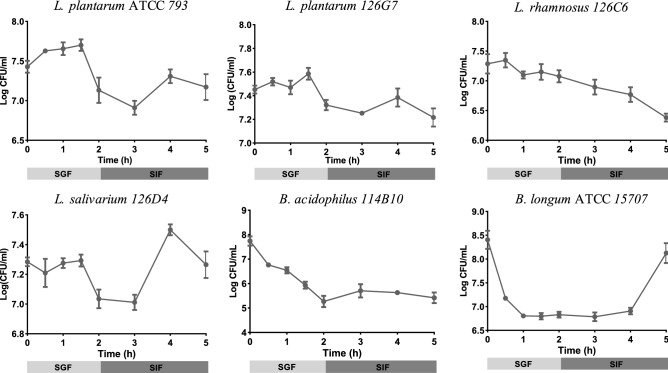
Testing probiotic integrity in simulated conditions. Gut bacteria isolated from healthy human fecal matter were verified for their probiotic activity by testing acid and bile tolerance in 2 h of simulated gastric fluid (SGF) and subsequently, 3 h in simulated intestinal fluid (SIF), respectively. Enumeration of *Lactobacillus* species was conducted with serial plating on Man-Rogosa-Sharpe (MRS) media under aerobic conditions while *Bifidobacteria* species were quantified on MRS media supplemented with cysteine in anaerobic conditions. Each time-point represents *n* = 3 independent trials +/− SEM. Variability over time was conducted with a one-way ANOVA using Kruskal Wallis analysis where *p < 0.05. −

To establish the ability of the ABIOME to support metabolic activity of the stabilized microbiota, the ABIOME was treated with a variety of novel probiotics and a grape-derived prebiotic previous verified to produce bioactive metabolites with therapeutic benefits^5^ in a manner that depends on the gut microbiota^6^. The prebiotic is a Botanical Derived Polyphenol Preparation (BDPP) containing grape seed polyphenol extract (1.0% *w/v*), concord grape extract powder (5.0% *w/v*) and resveratrol (1.0% *w/v*). The probiotics to be tested include two commercially available probiotics, *L. plantarum* ATCC 793 (Lp793) and *B. longum* ATCC 15707 (BL15707) and four experimental probiotics derived from healthy human fecal matter and identified using LEA-Seq^6^. The bacteria to be included in the probiotic characterization are *L. plantarum* 126A7 (Lp126A7), *L. rhamnosus* (Lr126C6), *L. salivarium* (Ls126D4), *B. adolescentis* 115B10 (Ba114B10) and *L. salivarius* 126D4. To be classified as a probiotic, these untested bacteria must be acid and bile resistant and demonstrate beneficial physiological effects. Acid and bile tolerance was tested by subjecting the isolated bacterial strains to simulated gastric fluid (SGF) and simulated intestinal fluid (SIF) emulating their movement through the GI tract. Lp793 showed resilience to SGF, but a slight sensitivity to bile. In contrast, BL15707 showed a small sensitivity to acid with an apparent tolerance to bile as its populations reestablished over the 5 h in SIF. The novel Lp126G7 responded similarly to Lp793 as it was generally resistant to acid with a slight sensitivity to bile. Lr126C6 demonstrated resilience to both acid and bile with only slight decrease in levels in both conditions while Ls126D4 was resistant to both SGF and SIF. Finally, Ba114B10, like BL15707, demonstrated a small sensitivity to acid and resistance to bile (Fig. 4).

**Figure 5:**
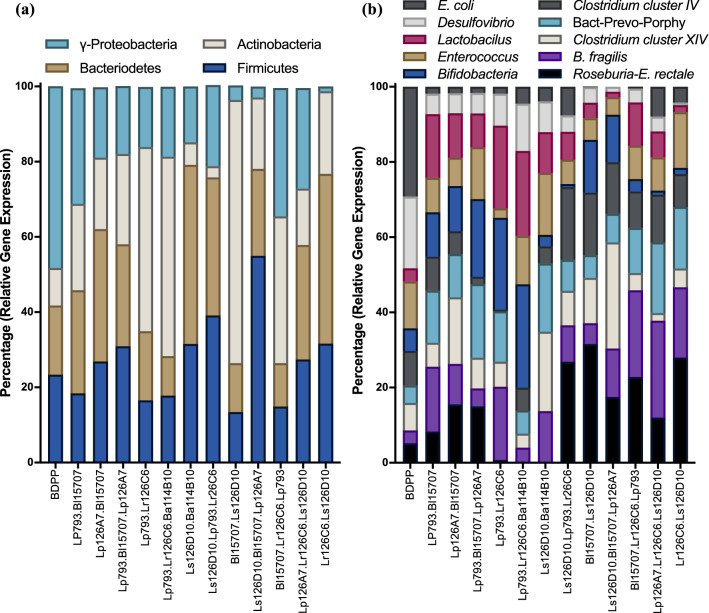
Relative gut microbiome population variations in the abiome following probiotic treatment. A representation of select gut microbiome (**a**) phyla and (**b**) genera from ABIOME inoculated with human fecal matter and subsequently treated with combinations of novel probiotics is presented. Populations were calculated using 16S qPCR rRNA analysis of the *V1-V3* region of each of the groups and represented using the 2^−ΔΔCT^ method relative to a reference sample and the total amount of bacteria as determined by the 27F/519R primer set. Across combination, the relative gene expression is shown as a percentage of the total relative populations. Each sample represents *n* = 3 trials and between-group analysis was calculated using the Mann Whitney test.

Twelve combinations of the six tested probiotic bacteria (Suppl. Table 2) were tested in the presence of the BDPP prebiotic for their effect on the composition of the gut microbiota and the production of a host of microbiota-derived polyphenolic metabolites (Fig. 5). Compared to BDPP alone, each of the probiotic combinations elicited an independent effect on the overall gut microbiota architecture. *E. coli* and γ-Proteobacteria levels in the BDPP alone group were high with a relative gene expression of 2.91 and 2.32, respectively; values that were consistently reduced by all combinations of probiotics. A similar trend with the *Desulfovibrio* group was observed, with a relative expression of 1.91 in the BDPP-alone group, which was significantly higher than in any of the probiotic-treated groups. Both combinations 4 and 5 showed a very low amounts of butyrate-producing bacteria including *C. leptum, C. coccides* and *Roseburia-E. rectale* groups, which was reflected by a low amount of Butyryl-CoA CoA-transferase gene expression. *Bifidobacteria* spp. expression was significantly lower in combinations 7, 11 and 12, which coincidently were not exogenously supplemented with *Bifidobacteria* probiotics. Interestingly, this trend did not apply to combination 4, which, while not containing exogenous *Bifidobacteria* species, still held a significant amount of the genera.

**Figure 6:**
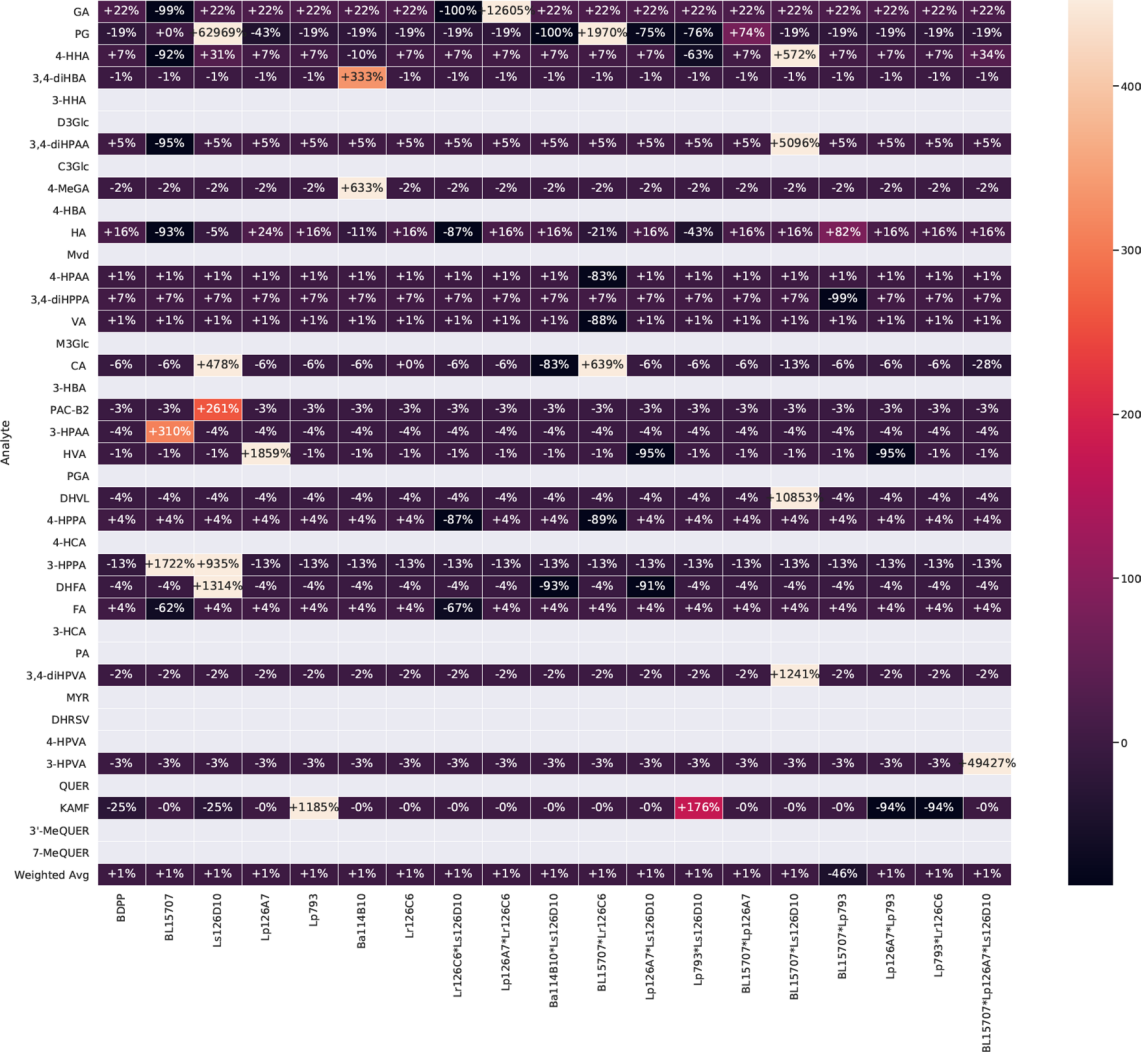
Predicted synergisms in metabolite production between probiotics using the MARS algorithm. Metabolite production from the polyphenolic precursor BDPP after exposure to 5 days of 12 probiotic combinations was calculated with a UPLC-QqQ/MS. Each row represents an individual metabolite, while each column represents a unique probiotic or combination of probiotics. For each metabolite, the model coefficients are represented as percentages. Rows masked in grey indicate metabolites for which production was not affected by the presence of probiotics. Gallic acid (GA), pyrogallol (PG), 4-hydroxyhippuric acid (4-HHA), 3,4-dihydroxybenzoic acid (3,4-HBA), 3-hydroxyhippuric acid (3-HHA), Delphinidin-3-O-glucoside (D3Glc), 3-(3,4-dihydroxyphenyl)propionic acid (3,4-diHPPA), cyanidin-3-glucoside (C3Glc), 4-O-methylgallic acid (4-MeGA), 4-hydroxybenzoic acid (4-HBA), hippuric acid (HA), malvidin (Mvd), 4-hydroxyphenylacetic acid (4-HPAA), 3,4-dihydroxyphenylacetic acid (3,4-diHPAA), vanillic acid (VA), malvidin-3-glucoside (M3Glc), caffeic acid (CA), 3-hydroxybenzoic acid (3-HBA), proanthocyanidin dimer B2 (PAC-B2), 3-hydroxyphenylacetic acid (3-HPAA), homovanillic acid (HVA), phloroglucinaldehyde (PGA), 5-(3′,4′-dihydroxyphenyl)-γ-valerolactone (DHVL), 3-(4-hydroxyphenyl)propionic acid (4-HPPA), 4-hydroxycinnamic acid (4-HCA), 3-(3-hydroxyphenyl)propionic acid (3-HPPA), dihydroferulic acid (DHFA), ferulic acid (FA), 3-hydroxycinnamic acid (3-HCA), phenylacetic acid (PA), 5-(3,4-dihydroxyphenyl)valeric acid (3,4-diHPVA), myricetin (MYR), dihydroresveratrol (DHRSV), 5-(4-hydroxyphenyl)valeric acid (4-HPVA), 5-(3-hydroxyphenyl)valeric acid (3-HPVA), quercetin (QUER), kaempferol (KAMF), isorhamnetin (3′-MeQUER), rhamnetin (7′-MeQUER).

To evaluate how probiotic combinations impact gut-microbiota derived polyphenolic metabolite production from the BDPP prebiotic, the ABIOME supernatant from each of the probiotic combinations was analyzed for polyphenolic metabolite production using UPLC-QqQ/MS. The MARS algorithm was then employed to predict if single probiotics or a combination formula may have synergistic interactions on metabolite production. Briefly, the impact of each individual probiotic on polyphenol production was measured, then the predictive ability of available probiotic combinations was assessed. Probiotics or combinations thereof that did not predict polyphenol levels were removed from the final model. The coefficients for each term in the model were normalized to relative percentages, with positive and negative percentages indicating that the term increased or decreased, respectively, polyphenol production in relation to the reference polyphenol level (Fig. 6). As indicated by the blank rows, some metabolites’ production were not affected by the presence of probiotics, including 3-HHA, D3Glc, C3Glc, 4-HBA, Mvd, M3Glc, 3-HBA, PGA, 4-HCA, 3-HCA, PA, MYR, DHRSV, 4-HPVA, QUER, 3′-MeQUER or 7′-MeQUER. The absence of an effect suggests that none of the tested probiotics or their combinations have a synergistic impact on metabolite production. However, an alternative explanation is that all the probiotics (or their combinations) produce a similar amount of the given metabolite. Importantly, a negative association does not necessarily represent a maladaptive effect of the probiotics as many of the listed metabolites can act as precursors for the production of more bioactive metabolic products.

All of the individual probiotics, except Lr126C6, demonstrated some beneficial effect on isolated metabolites. The single probiotic effect indicates that this probiotic alone, in the context of the adapted commensal gut microbiota, can increase levels of certain metabolites. Ls126D10 significantly increased PG, CA, PAC-B2, 3-HPPA and DHFA while BL15707 upregulated 3-HPAA and 3-HPPA at the expense of 4-HHA and FA. Both of the *L. plantarum* species elicited unique effects on metabolite production with Lp793 upregulating KAMF by 1185% and Lp126A7 acting on the distinctly different HVA, upregulating it by 1859%. Ba114B10 was uniquely able to upregulate 3,4-diHBA and 4-MeGA, while BL15707 alone increased the production of 3-HPAA and 3-HPPA by 310% and 1722%, respectively.

As expected, several of the probiotics worked together synergistically for the production of specific metabolites. The most drastic synergism was potentially between BL15707 and Ls126D10. Together, these probiotics elevated the production of 4-HHA by 572%, 3,4-diHBA by 5096%, DHVL by 10,853% and 3,4-diHPVA by 1241%. In contrast, BL15707 together with Lp126A7 had little synergistic advantage except a 74% increase in PG production. Lp793 together with Ls126D10 elevated the production of KAMF, though not as high as Lp793 alone, indicating that Ls126D10 may be using the KAMF product produced by Lp793 to generate downstream metabolites. Other minor interactions were observed between BL15707 with Lp793, Lp126A7 with Lp793 and Lp793 with Lr126C6. One tertiary interaction between BL15707, Lp126A7 and Ls126D10 created a predicted 49,427% increase in 3-HPVA, which was a unique increase compared to all the single and double predicted associations.

## Discussion

The gut microbiota has emerged as a key player in numerous diseases from irritable bowel syndrome and colon cancer, to neurological disorders like Alzheimer’s disease and Major Depressive Disorder^3^. One major hurdle for the development of therapeutic probiotics is the lack of humanized GI models that can be manipulated in controlled environments to study the influence of probiotics on gut microbiota communities and their metabolome. The gut microbiota is a significant player in the metabolism of nutritional elements, nutraceuticals, vitamins, minerals and dietary polyphenols making modelling its complexity a formidable task. To date, several in vitro models of the human GI tract have been developed, but are fundamentally limited by their size and/or complexity. In the current study, a novel state-of-the-art model of the human GI tract coined the ABIOME was designed and validated for its ability to sustain the human gut microbiota’s complexity and together with a machine learning algorithm, used to design optimized probiotic formulations with strong therapeutic potential.

The rate of fluid transfer, food composition and anaerobicity were the first major considerations in the design of the ABIOME’s program. The food and pancreatic solutions’ composition is essential to balance the breadth of commensal gut microbiota populations while the rate of fluid transfer must be optimized to promote constant microbial growth and the removal of waste products. To validate that the conditions could maintain a microbial culture, monocultures of two well-characterized probiotics, the aerotolerant *L. plantarum* and the anaerobic *B. longum*, were inoculated into the ABIOME over one day (three feeding cycles) and their sustained concentrations validated over 10 days. The greater challenge was to create an environment that sustained the full complexity of the commensal human gut microbiota. Several studies have shown that the gut microbiota media has a dramatic impact on the bacterial populations’ growth and metabolite activity^19,20^ as many of the fastidious microorganisms comprising the gut microbiota have uncharacterized nutritional requirements. To control for this, a low-concentration carbohydrate media adapted from Kim et al.^20^ was used with additional prebiotic components and vitamin and mineral supplements as outlined by Goodman et al.^21^ and gastric mucins. This hybrid media was designed to closely emulate the physiological conditions faced by the gut microbiota while supporting growth of all the major groups and preventing over expansion of opportunistic pathogens. Using this optimized media, a battery of both viable colonies and 16S rRNA *V1-V3* qPCR identified groups were maintained in the ABIOME over a period of 10 days, with an overall stabilization time of 5–6 days. The culture-dependent viability counting and qPCR analysis represent complementary approaches to examining the microbial culture diversity. The culture-based method allows living groups of bacterial species to be assessed, though has the caveat that the overall composition may not be fully represented and it cannot discriminate between different bacterial groups due to similar dependences on culture conditions. To control for this restriction, the culture-dependent method was coupled with a qPCR analysis that allows a finer level of discrimination to be accomplished. As expected, most major groups displayed a large inter-trial variability over the first few days of stabilization reflecting the differences in the composition of the donor samples. Some groups have tried to circumvent this issue by pooling multiple fecal donations, however using this technique, while the biodiversity of the pooled samples was slightly higher^22^, the overall functionality was not influenced^22,23^. An essential component of the stabilization period is the narrowing of the error bars over the first 5 days demonstrating that a new gut microbiota population “constant” can be established in the ABIOME, despite the initial variation, facilitating serial experiments over time. Based on the viable cell counts, the ABIOME can maintain both aerobic and anaerobic cultures with a minimal loss over time. However, it has been reported that the anaerobic bacteria outnumber the aerobic species by a factor of 2–3 fold^24^, which was not reflected in the current model. It is possible that the time from defecation to culture, which averaged under 30 min, was sufficient to reduce anaerobic populations. In addition, despite the advancing techniques in anaerobic culture, it has been reported that only 56% of human gut microbiota can be cultured^25^ inherently limiting the utility of ex vivo modelling. Finally, one caveat that should be mentioned in the single-bioreactor design is the ability of the ABIOME to only model one gastrointestinal compartment at a time. Unlike the SHIME model which has three dynamic bioreactor units with individual control of their respective dynamics (i.e. pH), the ABIOME can only emulate one of these compartments and its complexity may be altered without the gradually changing GI conditions. That being said, the ABIOME could be adapted to emulate either the ascending or transcending compartments too, by varying the pH of the bioreactor and the proportion of food to pancreatic solution that is released into the bioreactor.

Some of the obligatory anaerobic groups including the Clostridium clusters IV and XIVa had reduced populations over time. This could be from both the lag in defecation to culture time, or from the lack of a mucosal interface to facilitate their growth. Surface-adherent and luminal microbial populations, like the Clostridium clusters, have distinct roles within the GI ecosystem due to the physical differences in their microenvironments and proximity to the epithelium. Mucosal gut microbiota populations reside in a thick glycosylated mucin layer in a unique anoxic–oxic interface giving them distinct adherent and aerotolerant features^13,26^. There are many different types of glycans associated with the mucin layer creating distinct physiological niches that are consequently inhabited by specific microbial populations^27^. As such, there is also a proximal–distal increasing gradient of sialic acid mucins from the ileum to the colon, defining longitudinally different microbial niches characterized by the pH and composition of mucin-associate glycans^28^. In addition, the close proximity of the mucosal populations to the gut epithelium facilitates nutrient exchange and regulation of host innate immunity^29^. Like the M-SHIME, the ABIOME could be adapted to include a mucosal compartment with the addition of mucin-coated microcosms^9^, which would increase the viability of some of the strictly anaerobic groups that reside in the mucosal layer. Mucins can also serve as a growth substrate for several butyrate-producing Firmicutes, which may skew the populations of Firmicutes in the ABIOME. This includes the Clostridium cluster XIVa group accounting for almost 60% of the mucin-adhered microbiota in the M-SHIME^30^ and the *Roseburia* spp. and *E. rectale* that specifically colonize mucins. *Lachnospiraceae. Ruminococcaceae, Eubacteriaceae, Lactobacillaceae, Clostridiaceae* and *Erysipelotrichaceae* families were also found enriched in the mucin compartments of the similar TWINSHIME^31^. This loss of Clostridium clusters IV and XIV in the ABIOME could account for the loss of functional butyrate production observed with the quantification of the butyryl-CoA CoA gene. The addition of mucin microcosms into the ABIOME would introduce a new complexity for the collection of samples as intestinal biopsies would be necessary as mucosal bacterial populations would not be found in sufficient quantities in defecated fecal matter.

Despite these limitations, the ABIOME is a superior tool to understand the functional dynamics of the human gut microbiota and to monitor its response to both therapeutic and harmful challenges. In the context of the BDPP prebiotic supplemented microbial media, treatment with different combinations of probiotics elicited significant variations in the 16S qPCR detection of the gut microbiota species. Within the luminal compartment, probiotics can alter the composition and function of the gut microbiota by competition for or production of nutritional substrates, facilitating the bioconversion of nutritional substrates into subsequent growth substrates or even producing antimicrobial compounds that suppress the growth of other microorganisms^32,33^. In response to the probiotic combinations in the ABIOME, most of the changes were adaptive reductions in the opportunistic γ-Proteobacteria group and *E. coli* with an concurrent increase in beneficial butyrate-producing bacteria including the *Roseburia-E. rectale* group and Clostridium clusters IV and XIVa. The variations in the gut microbiota may not fully represent what would happen in vivo as the interaction of the probiotics with the epithelial barrier can alter the innate immune cells in the intestinal epithelia and hence, some of the microbes in the lumen. Probiotics have also been shown to modulate key signaling pathways in the epithelial layer such as NF-κB and MAPK, which can alter the physiology and innate immune microenvironment in the lumen^34^.

The gut microbiota’s population fluctuations further complicate the potential synergisms of probiotic bacteria with the generation of bioactive metabolites as metabolite production is dependent on not only one or a combination of a few probiotic species, but the entire luminal milieu. The benefit of the current machine learning approach is that the MARS algorithm accounts for impact of the probiotic supplementation in the context of the changing microbiota constructing more accurate predictions of bioactive metabolite production. From the predicted triple synergism of BL15707, Lp126A7 and Ls126D10, 3-HPVA production was elevated by almost 50,000%. 3-HPVA is one of the main colonic metabolites from the flavan-3-ols, such as catechin and epicatechin, responsible for their biological activity including their ability to reduce inflammation and increase neurite number and length^35^. In addition, 3-HPVA correlates to *Clostridia* and *Actinobacteria* groups including the Clostridium cluster IV group, *F. plautii, Ruminococcus bromii* and others^35^. BL15707 and Ls126D10 also presented multiple synergisms for the production of 4-HHA, 3,4-diHPAA, DHVL and 3,4-diHPVA. 4-HHA is produced by the gut microbiota from naringenin polyphenols derived from both orange juice and grape extracts and has been shown to have a negative correlation with obesity^36^. 3,4-diHPAA is also a microbiota-derived metabolite of quercetin with beneficial effects against diabetes through the activation of the nuclear factor erythroid 2-related factor 2 (Nrf-2)^37^. Importantly, 3,4-diHPAA, also known as DOPAC, is a dopamine metabolite and can cross the blood–brain-barrier to elicit potent anti-depression effects^38^. DHVL is another interesting gut-derived metabolite produced downstream of proanthocyanidins. DHVL prevents tumor necrosis factor (TNF)α-stimulated adhesion of THP-1 human monocytic cells by downregulating vascular cell adhesion molecule-1 and monocyte chemotactic protein-1 and correspondingly, NF-κB transcription with potentially major implications for cardiovascular disease^39^. DHVL can also cross the blood–brain-barrier facilitating the anti-inflammatory gut-brain-axis connection^40^. Interestingly, BL15707 and Ls126D10 individually increased the production of different metabolites exemplifying the importance of cross-feeding and probiotic synergism. Both BL15707 and Ls126D10 increased the production of 3-HPPA, which falls along a different arm of microbial-derived quercetin metabolism. Regardless, 3-HPPA has a battery of its own metabolic activities including reduction of blood pressure and protection of cardiovascular health^41^. 3-HPPA derived from the microbial-derived fermentation of GSPE crosses the blood–brain-barrier in a dose-dependent manner to potentially interfere with the assembly of β-amyloid peptides into neurotoxic amyloid-β, giving it a key role in Alzheimer’s disease neuropathologies^42^.

Gut microbiota derived metabolite production from dietary polyphenols involves an integrated and complex series of interactions between supplemented probiotics and the ongoing changes to the autochthonous gut microbiota. Artificial models like the ABIOME are essential to understand how probiotics can manipulate the composition of the gut microbiota while providing a controlled environment to test metabolite production. Assessing these complex interactions with a machine learning algorithm allows dissection of the synergistic interactions. Predictions can also be made for potential new synergisms that may not have been tested due to the inherent restrictions of biological models with regards to time and expense. Overall, probiotic therapeutic development can be conducted in the ABIOME and optimized for metabolite production opening new therapeutic paradigms capitalizing on the human gut microbiota.

## Materials and methods

### ABIOME program

The ABIOME was adapted from the fully automated ambr250 modular benchtop microbioreactor (Sartorius, NY, USA) utilizing fully integrated microbial bioreactor vessels. Each bioreactor has a liquid capacity of 100–250 mL and is connected to five liquid reservoirs (2–125 mL reservoirs and 3–50 mL vessels) with one of the larger reservoirs connected to a chiller unit to maintain temperatures between 6 and 8 °C. Liquid transfer between the reservoirs and the bioreactor is controlled by a proprietary pump system integrated into the bioreactor units while removal of liquid waste material is controlled by an external peristaltic pump. The bioreactor units each contain pH and deoxygenation (DO) spot measures with a pH range of 2–8.5 and a DO range of 0–200%. Each bioreactor has the potential to be connected to three gasses into the headspace or sparged into the media. Each pump, pH calibration and DO sensor is dynamically controlled by a touch-screen user-interface that allows parallel control of 2–8 bioreactor units and real-time monitoring of experimental progress. Programmed set points and monitoring prevents critical errors from damaging the sensitive experimental setup (Supplementary Fig. 1a).

To model the human gastrointestinal (GI) tract, 150 mL of a faecal slurry containing 10% (*w/v*) strained fecal matter in a 2:1 mixture of microbial media to pancreatic solution was inoculated into the bioreactor. For this set of experiments, the fecal donor was consistent for all studies; a vegetarian with no history of antibiotic usage for the past 1 year. All contributions were voluntary and the subject provided informed consent before inclusion with approval from Mount Sinai’s Institutional Review Board. To model the anaerobic environment of the GIT, the bioreactor vessel was kept in an anaerobic environment (DO 0.5%) with continuous nitrogen sparging, while the bioreactor unit was maintained at 37 °C. The chilled reservoir was filled with an optimized microbial “food” source to support diverse gut microbiota growth, the other large reservoir was filled with a “pancreatic solution” while two of the small reservoirs filled with acid (hydrochloric acid, 0.5 M) and base (sodium hydroxide, 0.5 M) solutions to maintain the pH set-points. The gut microbiota “food”is an adapted media^20,21,43^ composed of starch 3.0 g/L, pectin 2.0 g/L, mucin type III 4.0 g/L, xylan 1.0 g/L, arabinogalactan 1.0 g/L, inulin 1.0 g/L, casein 3.0 g/L, peptone water 1.0 g/L, tryptone 0.5 g/L, yeast extract 3.0 g/L, glucose 0.8 g/L, iron sulfate heptahydrate 0.005 g/L, sodium chloride 4.5 g/L, potassium chloride 4.5 g/L, potassium phosphate monohydrate 0.5 g/L, calcium chloride 0.8 g/L, cysteine 0.8 g/L, hemin 0.05 g/L, resazurin 1 mL of 0.01% solution, vitamin solution 1 mL (ATCC MD-VS) and trace mineral solution 1 mL (ATCC MD-TMS) and kept at pH 2.0 emulating the stomach component. The pancreatic solution was composed of sodium bicarbonate 12.0 g/L, oxgall 6.0 g/L and pancreatin 1.0 g/L. Emulating the environment found in the small intestine and typical pH changes due to the emptying of the stomach and the pancreas, the microbial media is (20 mL) and pancreatic solution (10 mL) were delivered simultaneously thrice daily (9:00, 17:00 and 1:00) into the bioreactor unit followed by 2 h incubation at pH 7.75. To model the conditions in the distal colon, the area of most diverse bacteria culture, the bioreactor’s pH is slowly ramped over 30 min to pH 6.8 for an additional 6 h to complete the 8 h feeding cycle. To recycle “waste” from the bioreactor, the external peristaltic pump is programmed to remove 35 mL of solution into an external container. Every trial conducted with the ABIOME was rigorously monitored using the real-time results viewer integrated into the software. A representative profile of typical pH, temperature, volume and liquid release is shown in Supplemental Figure 1.

### Single bacteria survival

Bacteria (*Bifidobacteria longum* ATCC 15707 or *Lactobacillus plantarum* ATCC 793) were grown aerobically for 18 h in Man-Rogosa-Sharpe (MRS) media (Sigma) or anaerobically for 36 h in MRS supplemented with 0.5 g/L cysteine, respectively. Anaerobic growth was achieved using an anaerobic chamber (Don Whitley DG250) connected to an anaerobic external gas (80% nitrogen, 10% carbon dioxide, 10% hydrogen) with nitrogen sparging for the cuffs. The bacteria was inoculated into the microbial media such that each feeding cycle would deliver 1 × 10^10^ CFU of bacteria into the bioreactor and the addition was allowed to occur for three cycles. Following inoculation, bacterial enumeration was conducted every 8 h for 3 days just before the next cycle of feeding. To count viable bacterial cells, inoculate was plated on MRS-agar plates (*L. plantarum*), grown aerobically for 18 h or MRS-cysteine-agar plates (*B. longum*), grown anaerobically for 36 h before colonies were counted.

### Agar selective plating

The ABIOME was inoculated as previously described. Samples were extracted everyday before the first feeding cycle, immediately transferred to an anaerobic chamber and serially diluted into a nutrient-rich media. Three dilutions (100 μL) were spread on agar-exclusion plates and grown according to the appropriate conditions described in Table 1.

**Table 1:** Selective agar composition for viable plate counts.

Bacterial group	Exclusion agar	Supplement	Growth conditions
All bacteria	Brain heart infusion agar	None	Aerobic, 18 h Anaerobic, 36 h
Gram negative	Eosin-methylene blue agar	None	Aerobic, 18 h Anaerobic, 36 h
Gram positive	Phenylethyl alcohol agar	None	Aerobic, 18 h Anaerobic, 36 h
*Bifidobacteria* spp.	BSC propionate agar base	Bifido selective supplement A (FD062)	Anaerobic, 36 h
*Lactobacillus* spp.	Man-Rogosa-sharpe agar	Vancomycin	Aerobic, 18 h
*Staphylococcus* spp.	Mannitol salt agar	None	Anaerobic, 36 h
*Enterococcus* spp.	Selective enterococcus agar	None	Anaerobic, 36 h
*Streptococcus* spp.	KF-streptococcus agar	Triphenyltetrazolium chloride	Anaerobic, 36 h
*Bacteroides *spp.	Bacteriodes bile esculin agar base	Bacteriodes selective supplement	Anaerobic, 36 h
Coliforms	Violet red bile agar	None	Anaerobic, 36 h
*Escherichia coli*	MacConkey agar	Sorbitol	Aerobic, 18 h

### Real-time PCR quantification

Using the same samples as the agar selective plating protocol, 5 mL of bacterial inoculate from the ABIOME was extracted using the syringe extraction port. Samples were centrifuged at 4000 rpm for 10 min at 4 °C to pellet the bacteria and the pellets were transferred to a − 80 °C until processing. Bacterial DNA was extracted using the FastDNA Spin kit for feces (Mp Bio, USA) with bead beating step preformed with the Bead Mill 24 Homogenizer (Fisher Scientific) using 5 rounds of 1 min homogenization steps at maximum speed and further following the manufacturer’s protocol. Bacterial DNA was quantified using a NanoDrop 2000 spectrophotometer (ThermoFisher, USA) and PCR amplified using primers 27F (GAGTTTGATCMTGGCTCAG) and 519R (GWATTACCGCGGCKGCTG). The PCR cycle was 1 cycle of 94 °C for 3 min, followed by 25 cycles of 94 °C for 45 s, 55 °C for 15 s and 72 °C for 30 s and cleaned up with the PureLink PCR purification kit (ThermoFisher, USA). Real time PCR was conducted using Power SybrGreen mastermix (ThermoFisher, USA) and the primers and melting temperatures in Table 2. Each of the primers, also mostly derived from the literature, were validated using the BLAST database compared to available 16S rDNA sequences to ensure specificity and inclusivity of the indicated group and were tested for sensitivity by conducting a relative standard curve with serial dilutions of a common amount of initial bacteria. Bacterial quantification was conducted using the 2^−ΔΔCT^ method relative to the total bacterial content and an arbitrary control sample for each group. In each case, the relative quantification is represented as the fold change compared to the arbitrary control sample so based on this method, the absolute quantification between groups cannot be assumed.

**Table 2:** Primer sequences for 16S rRNA V1–V3 region of major bacterial groups.

Bacterial group	Primer (5′–3′)	Anneal temp (°C)	
Firmicutes	F: TGAAACTYAAAGGAATTGACG R: ACCATGCACCACCTGTC	57	^44^
Bacteroidetes	F: CRAACAGGATTAGATACCCT R: GGTAAGGTTCCTCGCGTAT	56	^45^
Actinobacteria	F: TACGGCCGCAAGGCTA R: TCRTCCCCACCTTCCTCCG	55	^46^
γ-Proteobacteria	F: TCGTCAGCTCGTGTYGTGA R: CGTAAGGGCCATGATG	57	^47^
*Clostridium* cluster XIV	F: AAATGACGGTACCTGACTAA R: CTTTGAGTTTCATTCTTGCGA	59	^48^
*Lactobacillus*	F: AGCAGTAGGGAATCTTCCA R: CACCGCTACACATGGAG	60	^49^
*Enterococcus*	F: CCCTTATTGTTAGTTGCCATCATT R: ACTCGTTGTACTTCCCATTGT	61	^50^
*Bacteroides–Prevotella–Porphyromonas* group	F: GGTGTCGGCTTAAGTGCCAT R: CGGACGTAAGGGCCGTGC	62	^51^
*Desulfovibrio*	F: GGTACCTTCAAAGGAAGCAC R: GGGATTTCACCCCTGACTTA	62	^50^
*Bifidobacteria*	F: TCGCGTCYGGTGTGAAAG R: CCACATCCAGCRTCCAC	56	^50^
*Roseburia* spp.–*E. rectale*	F: GCGGTRCGGCAAGTCTGA R: CCTCCGACACTCTAGTMCGAC	63	^52^
Butyryl-coenzyme A-CoA transferase	F: GCIGAICATTTCACITGGAAYWSITGGCAYATG R: CCTGCCTTTGCAATRTCIACRAANGC	64	^18^
*B. fragilis*	F: ATAGCCTTTCGAAAGRAAGAT R: CCAGTATCAACTGCAATTTTA	57	^53^
*Escherichia coli*	F: CATGCCGCGTGTATGAAGAA R: CGGGTAACGTCAATGAGCAAA	64	^54^
*Clostridium coccides* group	F: AAATGACGGTACCTGACTAA R: CTTTGAGTTTCATTCTTGCGAA	59	^48^
*Clostridium leptum* group	F: GCACAAGCAGTGGAGT R: CTTCCTCCGTTTTGTCAA	54	^55^

### Probiotic testing

Bacteria strains were previously isolated^56^ from human fecal samples obtained from a stool biobank generated from Institutional Review Board approved clinical studies in accordance with the policies from Mount Sinai. Select strains of interest will be cultured anaerobically in an adapted universal nutrient-rich media^20^: brain–heart infusion 37 g/L, yeast extract 5 g/L, xylan 1 g/L, fructose 1 g/L, galatose 1 g/L, cellobiose 1 g/L, maltose 1 g/L, sucrose 1 g/L, arabinose 0.5 g/L, l-cysteine 0.5 g/L, malic acid 1 g/L, sodium sulfate 2 g/L, *N*-acetylglucosamine 0.5 g/L, 0.1 M potassium phosphate, 1 mL/L resazurin 0.25 mg/mL, 1 mL/L menadione 1 mg/mL, 1 mL/L hematin 1.2 mg/mL, 100 μL/L iron sulfide 4 mg/mL, 1 mL/L calcium chloride 0.8 mg/mL and Tween-80 0.5 mL/L at pH 7.2. The novel gut bacteria were tested for their probiotic efficacy based on their in vitro culturability and resilience to a 2 h incubation in simulated gastric fluid (0.2 M HCl, 0.2 M KCl and 10 U/ml pepsin at pH 2.5) and 8 h incubation in simulated pancreatic solution (bile salts, NaHCO_3_ and pancreatin at pH 6.8). Probiotics were sampled every 30 min while incubating in the SGF and every hour in the SIF for a total of 5 h and plated on the nutrient-rich media with agar plates anaerobically for 36 h.

### Metabolite detection

Identification and quantification of 44 phenolic compounds including parent BDPP polyphenols and phenolic acid metabolites (PAMs) in samples fermented with the 12 different probiotic combinations were conducted using UPLC-QqQ-MS/MS as per a previously reported method^57^ with modifications. Briefly, the samples were allowed to thaw on ice and conditioned to room temperature before processing. After vortexing, 500 μL of bacterial broth were acidified with 100 μL of 4 M HCl solution and spiked with 50 μL of *trans*-cinnamic acid-d_7_ and 4-Hydroxybenzoic acid-d_4_ (2 µg/mL each) as internal standards (I.S.), and mixed well. The mixture was then extracted with ethyl acetate (500 μL), followed by vortexing vigorously for 1 min, and centrifuged at 8000*×g* for 5 min using a micro-centrifuge. The upper organic phase (~ 450 μL) was transferred to a 1-dram glass vial. After two more extractions with ethyl acetate (500 μL) each, the combined supernatants were mixed with 10 μL of 2% ascorbic acid and dried under a gentle stream of nitrogen. The residue was reconstituted in 1000 μL of 60% methanol containing 0.1% formic acid and centrifuged at 16,000×*g* for 10 min. For each sample extract, 5 μL was injected twice into an Agilent 1290 Infinity II UHPLC (Agilent Technology, Palo Alto, CA, USA) system interfaced with an Agilent 6470 Triple Quadrupole Mass Spectrometer for analysis. Chromatographic separation was achieved using a Waters Acquity UPLC BEH C18 column (2.1 × 50 mm, 1.7 μm) (Milford, Massachusetts, USA) equipped with a Waters VanGuard Acquity C18 guard column (2.1 × 5 mm, 1.7 μm). The elution gradient started at 96% phase A and 4% phase B, held for 1.5 min before increasing % phase B to 12% in 12.5 min, to 90% in 1 min and held for another 2 min, and then returned to initial conditions in 1 min. The column was equilibrated for another 5 min before the next injection. Mass spectral data were acquired under both polarities and dynamic multiple reaction monitoring (dMRM) mode. Identification of phenolic analytes was achieved by comparing their parent-product ion pair transitions and retention times with those of the authentic standards. Quantitation was achieved with calibration curves established using the analyte-to-IS peak area of quantifier ions. Details of phenolic compounds included in the LC–MS analysis are listed in Supplementary Table 1.

### MARS algorithm

To predict which combinations of probiotics give the highest synergistic production of metabolites, we used multivariate adaptive regression splines (MARS)^58,59^ to build an in silico model that can predict the amount of metabolites using the concentration of probiotics and their interactions (Supplementary Figure 2). The input is $X\in {R}^{M},M=\#\text{probiotics}$ so that each $X$ can be represented by a vector of ${\left[{X}_{1},\dots ,{X}_{M}\right]}^{T}$ where ${X}_{i}$ is the concentration of the $i$-th probiotic; ${X}_{i}=0$ means the $i$-th probiotic is not used. The output is $Y \in R^{L} ,L = \# {\text{metabolites}}$ so that each $Y$ can be represented by a vector of ${\left[{Y}_{1},\dots ,{Y}_{L}\right]}^{T}$ where ${Y}_{j}$ is the amount of the $j$-th metabolite produced. For simplicity, we will model each ${Y}_{j}$ separately (or use a weighted sum of all ${Y}_{j}$’s as the output). Our purpose is to build a set of functions ${f}_{j}\left(X\right):X\mapsto {Y}_{j}$. In MARS, such a function ${f}_{j}$ is a linear model of the basis functions:

1 $$f_{j} \left( X \right) = \sum\limits_{{k = 1}}^{K} {C_{k} B_{k} \left( X \right)}$$

where $h\left(x\right)=max\left(0,x\right)$ is a hinge function and ${t}_{k}$ is known as the knot of the hinge. The use of hinge functions makes the model nonlinear by fitting the data using a piece-wise regression and (4) allows us to incorporate interactions between two or more probiotics. The use of (2) introduces an intercept. The MARS algorithm will automatically search through the input space and find an optimal combination of probiotics and their interactions so that the generalization error is minimized. Once the model is fit, the coefficients ${C}_{k}$ can be interpreted as the importance of each term; and the knots ${t}_{k}$ can be used to determine the ranges of their effect. Thus, the MARS models make it easy to interpret the effects of different probiotics and their interactions.

### Statistics

Variations over time for the single bacteria survival, viability counts and 16S qPCR determination of gut bacteria species were determined using a one-way ANOVA with Kruskal–Wallis posthoc analyses. Between-group assessment of gut microbiota populations in response to the combinations of probiotics was assessed with Mann–Whitney test for non-parametric samples. All statistical analyses were conducted in GraphPad Prism version 8.4.0 for windows (La Jolla, USA).

## Supplementary Information

Supplementary Information.

## Data Availability

All data generated or analysed during this study are included in this published article and its Supplementary Information files.

## References

[1] Yin R (2019). Gut microbiota, dietary phytochemicals, and benefits to human health. Curr. Pharmacol. Rep..

[2] Westfall S, Pasinetti GM (2019). The gut microbiota links dietary polyphenols with management of psychiatric mood disorders. Front. Neurosci..

[3] Westfall S (2017). Microbiome, probiotics and neurodegenerative diseases: Deciphering the gut brain axis. Cell Mol. Life Sci..

[4] O'Toole PW, Marchesi JR, Hill C (2017). Next-generation probiotics: The spectrum from probiotics to live biotherapeutics. Nat. Microbiol..

[5] Wang J (2018). Epigenetic modulation of inflammation and synaptic plasticity promotes resilience against stress in mice. Nat. Commun..

[6] Frolinger T (2018). Dietary polyphenols promote resilience against sleep deprivation-induced cognitive impairment by activating protein translation. FASEB J..

[7] Pearce SC (2018). Intestinal in vitro and ex vivo models to study host–microbiome interactions and acute stressors. Front. Physiol..

[8] Molly K, Vande Woestyne M, Verstraete W (1993). Development of a 5-step multi-chamber reactor as a simulation of the human intestinal microbial ecosystem. Appl. Microbiol. Biotechnol..

[9] Van den Abbeele P (2012). Incorporating a mucosal environment in a dynamic gut model results in a more representative colonization by lactobacilli. Microb. Biotechnol..

[10] McDonald JAK (2013). Evaluation of microbial community reproducibility, stability and composition in a human distal gut chemostat model. J. Microbiol. Methods.

[11] Venema K, van den Abbeele P (2013). Experimental models of the gut microbiome. Best Pract. Res. Clin. Gastroenterol..

[12] Bein A (2018). Microfluidic organ-on-a-chip models of human intestine. Cell Mol. Gastroenterol. Hepatol..

[13] Shin W (2019). A robust longitudinal co-culture of obligate anaerobic gut microbiome with human intestinal epithelium in an anoxic-oxic interface-on-a-chip. Front. Bioeng. Biotechnol..

[14] Mukhtar K, Nawaz H, Abid S (2019). Functional gastrointestinal disorders and gut-brain axis: What does the future hold?. World J. Gastroenterol..

[15] Luo Y-M (2018). A machine learning model based on initial gut microbiome data for predicting changes of Bifidobacterium after prebiotics consumption. Nan Fang Yi Ke Da Xue Xue Bao.

[16] Forbes JD (2018). A comparative study of the gut microbiota in immune-mediated inflammatory diseases-does a common dysbiosis exist?. Microbiome.

[17] Gill SR (2006). Metagenomic analysis of the human distal gut microbiome. Science.

[18] Louis P, Flint HJ (2007). Development of a semiquantitative degenerate real-time pcr-based assay for estimation of numbers of butyryl-coenzyme A (CoA) CoA transferase genes in complex bacterial samples. Appl. Environ. Microbiol..

[19] Yousi F (2019). Evaluation of the effects of four media on human intestinal microbiota culture in vitro. AMB Express.

[20] Kim B-S, Kim JN, Cerniglia CE (2011). In vitro culture conditions for maintaining a complex population of human gastrointestinal tract microbiota. J. Biomed. Biotechnol..

[21] Goodman AL (2011). Extensive personal human gut microbiota culture collections characterized and manipulated in gnotobiotic mice. Proc. Natl. Acad. Sci. USA..

[22] Aguirre M, Ramiro-Garcia J, Koenen ME, Venema K (2014). To pool or not to pool? Impact of the use of individual and pooled fecal samples for in vitro fermentation studies. J. Microbiol. Methods.

[23] Human Microbiome Project, C (2012). Structure, function and diversity of the healthy human microbiome. Nature.

[24] Walsh CJ, Guinane CM, O'Toole PW, Cotter PD (2014). Beneficial modulation of the gut microbiota. FEBS Lett..

[25] Eckburg PB (2005). Diversity of the human intestinal microbial flora. Science.

[26] Espey MG (2013). Role of oxygen gradients in shaping redox relationships between the human intestine and its microbiota. Free Radical Biol. Med..

[27] Owen CD (2017). Unravelling the specificity and mechanism of sialic acid recognition by the gut symbiont *Ruminococcus gnavus*. Nat. Commun..

[28] Robbe C (2003). Evidence of regio-specific glycosylation in human intestinal mucins: Presence of an acidic gradient along the intestinal tract. J. Biol. Chem..

[29] Sonnenburg JL, Angenent LT, Gordon JI (2004). Getting a grip on things: How do communities of bacterial symbionts become established in our intestine?. Nat. Immunol..

[30] Van den Abbeele P (2013). Butyrate-producing Clostridium cluster XIVa species specifically colonize mucins in an in vitro gut model. ISME J..

[31] Liu L (2018). Establishing a mucosal gut microbial community in vitro using an artificial simulator. PLoS ONE.

[32] Spinler JK (2008). Human-derived probiotic *Lactobacillus reuteri* demonstrate antimicrobial activities targeting diverse enteric bacterial pathogens. Anaerobe.

[33] O'Shea EF, Cotter PD, Stanton C, Ross RP, Hill C (2012). Production of bioactive substances by intestinal bacteria as a basis for explaining probiotic mechanisms: Bacteriocins and conjugated linoleic acid. Int. J. Food Microbiol..

[34] Thomas CM, Versalovic J (2010). Probiotics-host communication: Modulation of signaling pathways in the intestine. Gut Microbes.

[35] Mena P (2019). Phenyl-γ-valerolactones and phenylvaleric acids, the main colonic metabolites of flavan-3-ols: Synthesis, analysis, bioavailability, and bioactivity. Nat. Prod. Rep..

[36] Palau-Rodriguez M (2015). Metabolomic insights into the intricate gut microbial-host interaction in the development of obesity and type 2 diabetes. Front. Microbiol..

[37] Carrasco-Pozo C, Gotteland M, Castillo RL, Chen C (2015). 3,4-Dihydroxyphenylacetic acid, a microbiota-derived metabolite of quercetin, protects against pancreatic β-cells dysfunction induced by high cholesterol. Exp. Cell Res..

[38] Valles-Colomer M (2019). The neuroactive potential of the human gut microbiota in quality of life and depression. Nat. Microbiol..

[39] Lee CC (2017). 5-(3',4'-Dihydroxyphenyl-γ-valerolactone), a major microbial metabolite of proanthocyanidin, attenuates THP-1 monocyte-endothelial adhesion. Int. J. Mol. Sci..

[40] Angelino D (2019). 5-(Hydroxyphenyl)-γ-valerolactone-sulfate, a key microbial metabolite of flavan-3-ols, is able to reach the brain: Evidence from different in silico, in vitro and in vivo experimental models. Nutrients.

[41] Najmanová I (2016). Flavonoid metabolite 3-(3-hydroxyphenyl)propionic acid formed by human microflora decreases arterial blood pressure in rats. Mol. Nutr. Food Res..

[42] Wang D (2015). Role of intestinal microbiota in the generation of polyphenol-derived phenolic acid mediated attenuation of Alzheimer's disease beta-amyloid oligomerization. Mol. Nutr. Food Res..

[43] Faith JJ (2013). The long-term stability of the human gut microbiota. Science.

[44] Li J (2019). Pollen reverses decreased lifespan, altered nutritional metabolism and suppressed immunity in honey bees (*Apis mellifera*) treated with antibiotics. J. Exp. Biol..

[45] Curtis MM (2014). The gut commensal Bacteroides thetaiotaomicron exacerbates enteric infection through modification of the metabolic landscape. Cell Host Microbe.

[46] Thévenot J (2013). Enterohemorrhagic *Escherichia col*i O157:H7 survival in an in vitro model of the human large intestine and interactions with probiotic yeasts and resident microbiota. Appl. Environ. Microbiol..

[47] Karamipour N, Fathipour Y, Mehrabadi M (2016). Gammaproteobacteria as essential primary symbionts in the striped shield bug, Graphosoma Lineatum (Hemiptera: Pentatomidae). Sci. Rep..

[48] Matsuki T (2002). Development of 16S rRNA-gene-targeted group-specific primers for the detection and identification of predominant bacteria in human feces. Appl. Environ. Microbiol..

[49] Su Y, Chen X, Liu M, Guo X (2017). Effect of three lactobacilli with strain-specific activities on the growth performance, faecal microbiota and ileum mucosa proteomics of piglets. J. Anim. Sci. Biotechnol..

[50] Rinttilä T, Kassinen A, Malinen E, Krogius L, Palva A (2004). Development of an extensive set of 16S rDNA-targeted primers for quantification of pathogenic and indigenous bacteria in faecal samples by real-time PCR. J. Appl. Microbiol..

[51] Pinto F, Medina DA, Pérez-Correa JR, Garrido D (2017). Modeling metabolic interactions in a consortium of the infant gut microbiome. Front. Microbiol..

[52] Ramirez-Farias C (2009). Effect of inulin on the human gut microbiota: Stimulation of *Bifidobacterium adolescentis* and *Faecalibacterium prausnitzii*. Br. J. Nutr..

[53] Carroll IM, Chang Y-H, Park J, Sartor RB, Ringel Y (2010). Luminal and mucosal-associated intestinal microbiota in patients with diarrhea-predominant irritable bowel syndrome. Gut Pathogens.

[54] Huijsdens XW (2002). Quantification of bacteria adherent to gastrointestinal mucosa by real-time PCR. J. Clin. Microbiol..

[55] Ji Y, Guo Q, Yin Y, Blachier F, Kong X (2018). Dietary proline supplementation alters colonic luminal microbiota and bacterial metabolite composition between days 45 and 70 of pregnancy in Huanjiang mini-pigs. J. Anim. Sci. Biotechnol..

[56] Britton GJ (2019). Microbiotas from humans with inflammatory bowel disease alter the balance of gut Th17 and RORγt(+) regulatory T cells and exacerbate colitis in mice. Immunity.

[57] Zhao D (2018). Development and validation of an ultra-high performance liquid chromatography/triple quadrupole mass spectrometry method for analyzing microbial-derived grape polyphenol metabolites. J. Chromatogr. B.

[58] Friedman JH (1991). Multivariate adaptive regression splines. Ann. Stat..

[59] Hastie T, Tibshirani R, Friedman JH (2009). The Elements of Statistical Learning: Data Mining, Interface, and Prediction.

