# Supplementary material for: Optimization of probiotic therapeutics using machine learning in an artificial human gastrointestinal tract
Source: Sci Rep. 2021 Jan 13;11:1067. doi: 10.1038/s41598-020-79947-y (PMC7806704; doi:10.1038/s41598-020-79947-y)
Supplement: Supplementary file 1 — Supplementary Information. [file 41598_2020_79947_MOESM1_ESM.pdf]

Supplementary Materials

Optimization of Probiotic Therapeutics using Machine Learning in an Artificial Human Gastrointestinal Tract

Susan Westfall[1], Francesca Carracci[1], Molly Estill[1], Danyue Zhao[3], Qing-li Wu[3], Li Shen[1], James Simon[3], Giulio Maria Pasinetti[1,2,*]

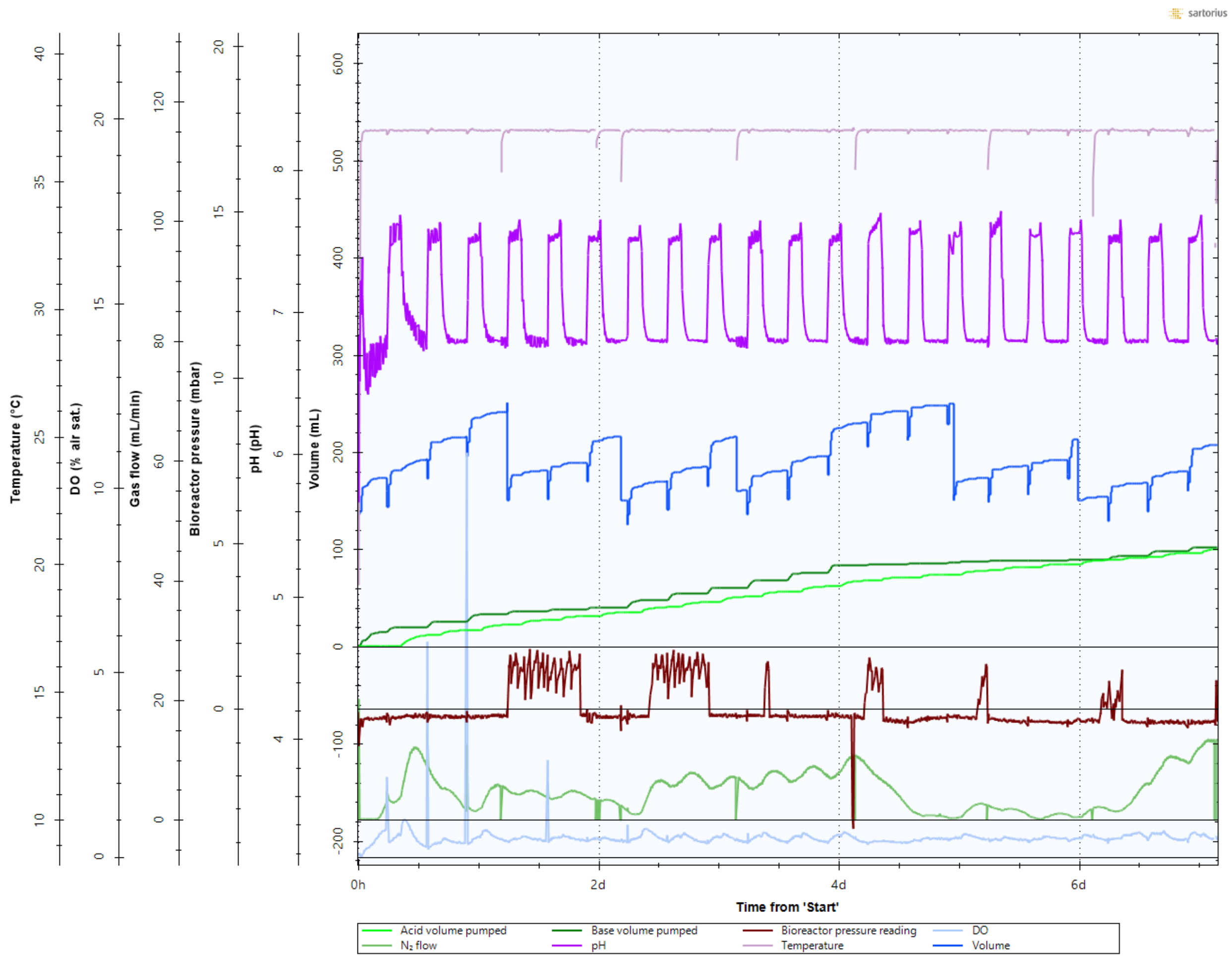

Supplementary Figure 1: Representative Conditions of the ABIOME. The ambr250 unit allows the real-time monitoring of all parameters inside the bioreactor allowing quality control to be conducted throughout each experiment. This includes temperature regulation (light purple), pH (pink), overall volume (blue), acid (dark green) and base (light green) volumes pumped, bioreactor pressure (brown), nitrogen gas flow (green) and deoxygenation (DO) level (light blue). The cyclic pH read out confirms the alterations between the feeding cycle and ileum incubation at pH 6.7, which is in line with the changes in volume associated with feeding. Small variations in the DO represent the small amount of oxygenation which is introduced into the bioreactor along with the food and pancreatic solution inputs while large dips in the total volume are due to manual corrections to maintain the bioreactor at 150 mL working volume.

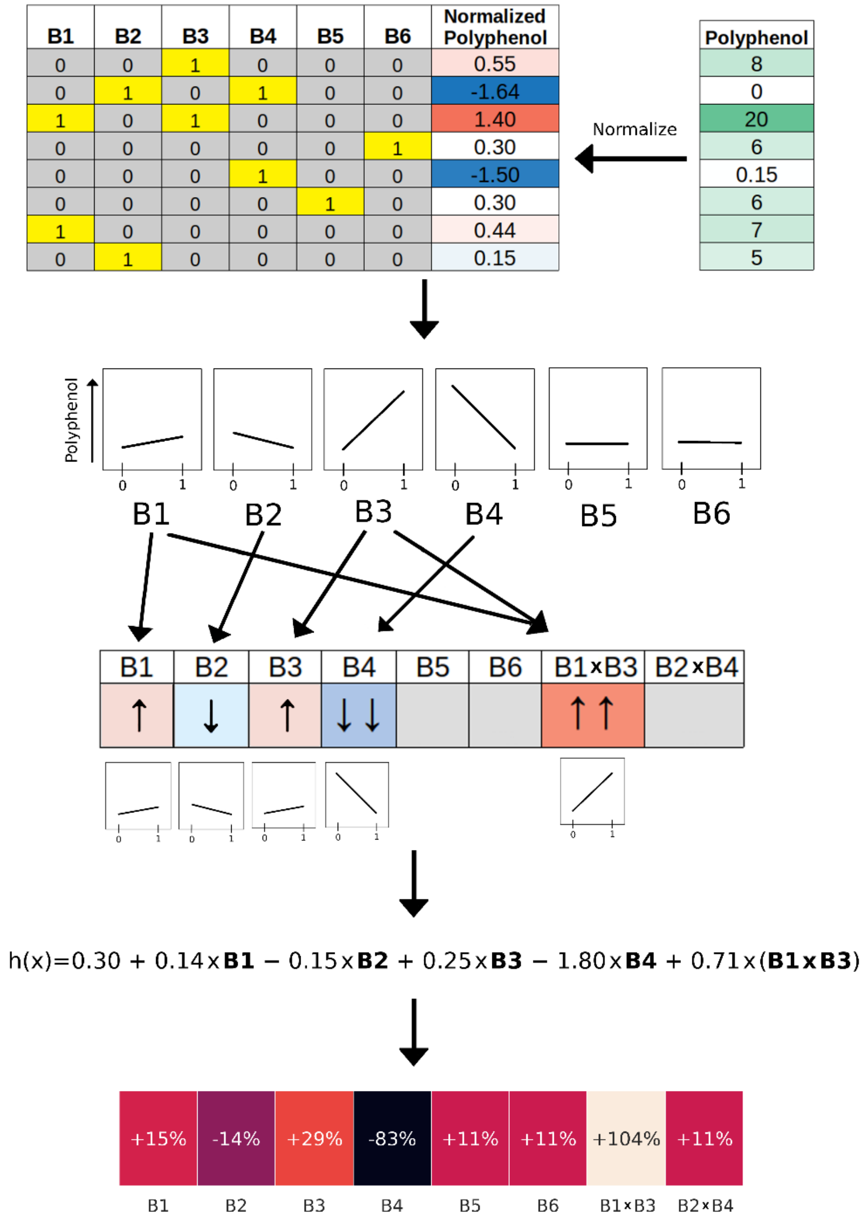

Supplementary Figure 2: Different bacterial combinations yield result in varying amounts of a given polyphenol. In the example matrix, the presence or absence of a bacterial strains is indicated with a "1" (in yellow), or "0" (in grey), respectively. Administration of the bacterial combinations shown produced a resulting polyphenol level, which was log-transformed and normalized for use in MARS. For each polyphenol, MARS first examines the response of the polyphenol level to the presence or absence (1 or 0) of the six individual bacterial strains (B1-6). In this example, B5 and 6 are not predictors of the polyphenol. The presence of B1 weakly predicts an increase in polyphenol, while the presence of B2 weakly predicts an decrease in polyphenol. Both B3 and B4 strongly predict polyphenol level, with the presence of B3 yielding an increase in polyphenol, and the presence of B4 yielding a decrease in polyphenol. MARS then investigates the possible interactions between the individual bacterial strains, which are limited

in this example dataset to the interaction of B1xB3 and B2xB4. Based on the influence of the individual formula terms on the polyphenol response, non-informative terms are removed from the formula. In this example, B5, B6, and the interaction of B2 and B4 (B2xB4) do not predict the polyphenol response, and are thus excluded from the final model. The model coefficients are then extracted and normalized.

Supplementary Table 1. Details of phenolic compounds included in the updated LC-QqQ MS/MS analysis.

No.	Compound Name	Abbreviation	Molecular Weight
1	Gallic acid	GA	170.12
2	Pyrogallol	PG	126.11
3	4-Hydroxyhippuric acid	4-HHA	195.17
4	Delphinidin-3-O-glucoside	D3Glc	465.39
5	3,4-Dihydroxybenzoic acid	3,4-diHBA	154.12
6	3-Hydroxyhippuric acid	3-HHA	195.17
7	3,4-Dihydroxyphenylacetic acid	3,4-diHPAA	168.15
8	4-O-Methylgallic acid	4-MeGA	184.15
9	Cyanidin-3-glucoside	C3Glc	449.39
10	4-Hydroxybenzoic acid	4-HBA	138.12
11	Catechin	C	290.26
12	Hippuric acid	HA	179.12
13	Malvidin	Mvd	331.30
14	4-Hydroxyphenylacetic acid	4-HPAA	152.15
15	3-(3,4-Dihydroxyphenyl)propionic acid	3,4-diHPPA	182.17
16	Vanillic acid	VA	168.15
17	Malvidin-3-glucoside	M3Glc	493.44
18	Caffeic acid	CA	180.16
19	3-Hydroxybenzoic acid	3-HBA	138.12
20	Proanthocyanidin dimer B2	PAC-B2	576.51
21	3-Hydroxyphenylacetic acid	3-HPAA	152.15
22	Homovanillic acid	HVA	182.17
23	Phloroglucinaldehyde	PGA	154.12
24	Epicatechin	EC	290.26
25	5-(3',4'-Dihydroxyphenyl)-γ-valerolactone	DHVL	208.21

26	3-(4-Hydroxyphenyl)propionic acid	4-HPPA	166.17
27	4-Hydroxycinnamic acid	4-HCA	164.17
28	O-Methyl epicatechin	Me-EC	304.30
29	Dihyroferulic acid	DHFA	196.21
30	3-(3-Hydroxyphenyl)propionic acid	3-HPPA	166.17
31	Ferulic acid	FA	194.18
32	3-Hydroxycinnamic acid	3-HCA	164.16
33	Phenylacetic acid	PA	136.15
34	5-(3,4-Dihydroxyphenyl)valeric acid	3,4-diHPVA	210.23
35	Myricetin	MYR	318.24
36	t-Resveratrol (-)	RSV	228.25
37	Dihydroresveratrol	DHRSV	230.26
38	5-(4-Hydroxyphenyl)valeric acid	4-HPVA	194.23
39	5-(3-Hydroxyphenyl)valeric acid	3-HPVA	194.23
40	Quercetin	QUER	302.26
41	Kaempferol	KAMF	286.23
42	Isorhamnetin	3'-MeQUER	316.27
43	Rhamnetin	7-MeQUER	316.27
44	4-hydroxybenzoic-2,3,5,6-d4	IS#1	142.12
45	*Trans*-cinnamic acid-d7	IS#2	155.16

Supplementary Table 2: Relative Gene Expression of Major Bacterial Groups in Response to Bacterial Combinations

**BDPP**			
**Combo 1**	Lp793	Bl15707	
**Combo 2**	Lp126A7	Bl1507	
**Combo 3**	Lp793	Bl15707	Lp126A7
**Combo 4**	Lp793	Lr126C6	
**Combo 5**	Lp793	Lr126C6	Ba114B10
**Combo 6**	Ls126D10	Ba114B10	
**Combo 7**	Ls126D10	Lp793	Lr126C6
**Combo 8**	Bl15707	Ls126D10	
**Combo 9**	Ls126D10	Bl15707	Lp126A7
**Combo 10**	Bl15707	Lr126C6	Lp793
**Combo 11**	Lp126A7	Lr126C6	Ls126D10
**Combo 12**	Lr126C6	Ls126D10	
